# Modelling the Structural Relationships Between COVID-19 Knowledge, Attitudes and Behaviours in Jordanian Undergraduates

**DOI:** 10.3390/ijerph23050590

**Published:** 2026-04-30

**Authors:** Saja Alnahar, Mahmoud Alquraan, Austen El-Osta

**Affiliations:** 1 The Institute of Public Health, The University of Jordan, Amman 11942, Jordan; 2 Self-Care Academic Research Unit (SCARU), Department of Primary Care & Public Health, School of Public Health, Imperial College London, London W12 0BZ, UK; a.el-osta@imperial.ac.uk; 3 College of Education, Al Ain University, Al Ain P.O. Box 64141, United Arab Emirates; mahmoud.alquraan@aau.ac.ae

**Keywords:** COVID-19, knowledge, health behaviour, attitude, path analysis, public health

## Abstract

**Highlights:**

**Public health relevance—How does this work relate to a public health issue?**

- This study emphasised the influence and importance of knowledge and attitude in promoting and sustaining protective behaviour during pandemics and public health crises.
- The empirical examination of the knowledge, attitude and behaviour model provided a deeper understanding of behavioural determinants of self-care practices, which are cornerstones of efficient public health responses during pandemics.

**Public health significance—Why is this work of significance to public health?**

- This study showed that knowledge alone is insufficient to trigger and sustain protective behaviours, as emerging evidence indicates that attitude mediates the promotion and encouragement of protective behaviours and practices. These findings highlight the importance of designing public health interventions that go beyond simple dissemination of knowledge and promotional materials.
- The reported difference between gender and academic disciplines underscored the structural and contextual inequities in health literacy and readiness to follow and adopt preventive self-care practices.

**Public health implications—What are the key implications or messages for practitioners, policy makers and/or researchers in public health?**

- For policy-makers, public health practitioners and health educators, the study’s results propose that efficient pandemic and health crises preparedness requires a comprehensive intervention that addresses aspects related to knowledge (cognitive), attitudes (affective) and contextual (structural) factors.
- For public health researchers, the current study offers analytical and theoretical support for the deployment of mediated behavioural models and theories, such as the KAB model, to understand the complex pathways through which knowledge and attitudes affect behaviour.

**Abstract:**

Background: Regulatory restrictions and mandates typically offer short-term behaviour guidance, whereas interventions to improve knowledge and attitudes could result in more sustainable behavioural changes. Health authorities implemented awareness campaigns to enhance public knowledge and attitudes regarding COVID-19. This study explored the interplay between knowledge, attitudes and behaviours related to COVID-19 among university undergraduate students in Jordan, aiming to inform public health initiatives and educational programmes. Methods: A cross-sectional survey targeting undergraduate students enrolled at Yarmouk University in Jordan was conducted between January and May 2021. Participants consented to complete an anonymised validated self-administered questionnaire to evaluate their understanding of COVID-19 symptoms, treatment and transmission and attitudes and behaviours towards preventive measures. Data were analysed using descriptive and inferential statistics and structural equation modelling to investigate the associations between knowledge, attitudes and behaviours. Results: A total of 1375 undergraduate students participated in the survey. Knowledge of COVID-19 was low among most participants, with only 1.3% demonstrating high knowledge. Conversely, 58.5% exhibited good behaviour, and 31.4% reported full compliance with recommended behaviours. Significant differences were found in knowledge, attitudes and behaviours across different faculty clusters, with health faculties showing superior knowledge and more positive attitudes. Female participants (66.3%) were more likely to engage in positive behaviours than males (*p*-value = 0.02). Structural equation model (SEM) analysis showed that knowledge significantly influenced attitudes, which affected behaviours, confirming the model’s validity. Conclusions: The study highlights the critical role of knowledge and attitudes in shaping COVID-19-related behaviours among university students. Significant variations in knowledge and attitudes across different academic disciplines highlight the need for tailored educational interventions. The analysis supports the theoretical model linking knowledge, attitudes and behaviours, emphasising the importance of improving knowledge and attitudes to drive behaviour change. The findings suggest that comprehensive health education programmes targeting cognitive and affective aspects are essential for effective public health responses during pandemics.

## 1. Background

The World Health Organisation (WHO) declared COVID-19 a pandemic in March 2020, advising countries to adopt a slow-and-stop strategy to reduce transmission and flatten the curve [1,2,3,4,5,6]. This strategy encouraged communication, promoted preventive measures, and discouraged mass gatherings and travel. Governments implemented strict social distancing measures, including partial or complete lockdowns [4,7]. The pandemic also significantly affected educational systems, leading to nearly total closures of schools, colleges, and universities. As of April 2020, nearly all educational institutions were shut down [8,9].

In addition to country- and institution-level measures, healthcare authorities were heavily involved in encouraging citizens to comply with authorities’ mandates and to adopt individual-level self-care practices to help limit the spread of severe acute respiratory syndrome coronavirus-2 (SARS-CoV-2), including handwashing and respiratory hygiene practices (RHPs) [10]. The latter encompass a variety of behaviours, such as covering the mouth and nose when coughing or sneezing, wearing face masks, and keeping a physical distance from other people [11,12]. Implementing these procedures was deemed essential to reduce the spread of COVID-19, particularly in public and communal areas [13]. Additionally, RHPs include avoiding direct contact with infected or at-risk individuals, staying at home when infected and enhancing ventilation in indoor places [14].

Evidence showed that public compliance with authoritative mandates and adherence to RHPs are influenced by several factors related to public health communications, cultural beliefs, societal norms and the perceived susceptibility and severity of COVID-19 [15,16,17]. Life in higher-education institutions (HEIs) necessarily involves social gatherings of students, educators, researchers and administrative staff. Accordingly, campuses with little or no social distancing measures could be hot spots for virus transmission. Therefore, the way students and staff behaved and their compliance with risk avoidance strategies, RHPs and social distancing measures played a central role in controlling and preventing the spread of COVID-19 in educational settings and communities.

Regulatory restrictions could guide behaviours, but their influence is often temporary and unsustainable [18,19]. On the other hand, interventions targeting knowledge and attitudes could lead to permanent and sustained behaviours [20,21,22]. Health authorities, while issuing behaviour-related mandates and guidelines, have also been actively involved in awareness campaigns primarily aimed at enhancing knowledge and health literacy among the general population and to cultivate positive attitudes toward RHPs and COVID-19 slow-and-stop procedures [23,24]. The importance of knowledge and attitudes in shaping sustainable behaviours cannot be overstated [23,24].

The current study was broadly guided by the Knowledge–Attitude–Behaviour (KAB) model, a frequently deployed model that investigates the interplay between attitude, knowledge and behaviour in healthcare settings. The model assumes that acquired knowledge would shape individuals’ attitudes and subsequently influence their health behaviour [25]. Despite being criticised for oversimplifying the behaviour-influencing factors, the model remains a valuable framework for hypothesis-driven research in health behaviour and promotion research. Over the past six years, several researchers have employed the KAB model to analyse population behaviours and responses to the COVID-19 pandemic. For instance, Azlan et al. (2020) in Malaysia and Limbu et al. (2020) in Nepal reported that higher COVID-19 knowledge triggered protective attitudes and adherence to preventative behaviours, such as respiratory hygiene practices [26,27]. This evidence highlights the significance of the KAB model in examining how undergraduate students’ knowledge and attitudes might influence their COVID-19-related behaviours and practices.

The current study explored the dynamics between knowledge, attitudes and behaviours toward COVID-19 of university undergraduate students in Jordan who were at a critical stage of social and academic development. By examining the associations between knowledge, attitudes and behaviours, this research sought to inform public health initiatives and educational programmes tailored to enhance the understanding and management of COVID-19, other pandemics and related public health emergencies.

**Figure 1:**
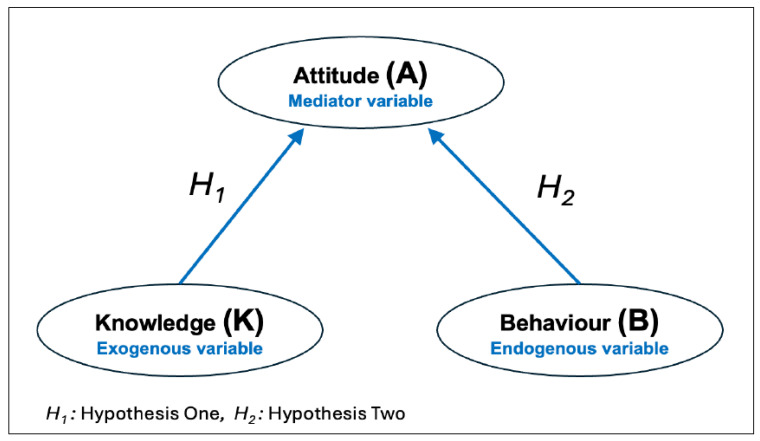
KAB model structure and proposed hypotheses; H1 (Hypothesis 1): knowledge has a significantly positive association with attitude; H2 (Hypothesis 2): attitude has a significant positive association with behaviour.

The aim of this study was to evaluate the knowledge, attitudes and behaviours [20] of university undergraduate students regarding COVID-19-related hygiene and precautionary practices. This was achieved by developing and validating a reliable instrument to assess knowledge, attitudes and behaviours (KAB) of HEI students. The tool consisted of three main variables: the endogenous variable (behaviours), the mediator variable (attitudes), and the exogenous variable (knowledge); Figure 1 illustrates the model.

## 2. Methods

### 2.1. Study Design

A cross-sectional study was conducted among undergraduate students attending Yarmouk University between January and May 2021. The study was carried out in three phases: Phase-1 aimed to develop a validated online tool that investigated undergraduate students’ knowledge, attitudes and behaviours related to COVID-19 RHPs. Phase-2 investigated the associations between knowledge, attitude and behaviour using a structural equation model approach, whereas Phase-3 assessed research participants’ knowledge, attitudes and behaviours regarding COVID-19 RHPs.

### 2.2. Tool Development and Validation

#### Items and Domains Development

The tool’s design was based on a thorough review of the available literature, guidelines and WHO statements [10,12], including relevant materials for other pandemics such as severe acute respiratory syndrome [28], avian influenza [29], the H1N1-2009 pandemic (swine flu) [30] and other viral respiratory infections such as influenza [31]. The relevant literature was reviewed to identify constructs and items related to the study variables, namely knowledge, attitudes, and behaviours. Elements identified were later grouped into constructs within three domains: knowledge (K), attitudes (A) and behaviours (B) based on Bish and Michie’s review [32], the WHO KAP Survey Development Guide [33] and the Flu Telephone Survey Template (FluTEST) [34].

### 2.3. Validation and Reliability Assessment

A panel of nine experts in epidemiology (n = 2), microbiology (n = 1), public health (n = 3) and health behaviour (n = 3) assessed the face and content validity of the first draft. The experts independently and subjectively assessed the drafted instrument’s readability, clarity of use terminology, language, relevance, compatibility between items and construct definition and scope and suitability and adequacy of the proposed scales. The reviewers were provided with a customised assessment form that addressed all assessment criteria.

The initial draft of the questionnaire instrument contained fifteen questions: four questions assessing knowledge, seven assessing attitudes, one assessing behaviour and three related to demographics and characteristics. Across the model-related domains, 134 items were included, while eight items were within the demographics. The tool’s reliability and internal consistency coefficients were determined in a pilot study with 78 participants who met similar eligibility criteria to the intended profile of research participants. The internal consistency of each construct was determined using Cronbach’s coefficient α analysis; Cronbach’s α of (0.60) and above was considered acceptable.

Based on the panel’s recommendations and the pilot study’s results, some items and questions were either merged, dropped, or rephrased for clarity to enhance the questionnaire instrument’s conceptual alignment and coherence.

### 2.4. Tool Description

The final refined tool consisted of fourteen questions grouped as follows: three questions related to participants’ demographics, three questions related to the knowledge domain (31 items grouped into three constructs), seven questions investigating attitudes (78 items grouped into seven constructs), and one question assessing behaviour (16 items in one construct). Table 1 details the constructs, constructs codes, items and scales used within each domain.

Data coding was domain- and construct-specific; for the knowledge (K) domain, any correct answer was awarded one point (+1), an incorrect answer was awarded minus one point (−1), and “do not know” answers were awarded zero points (0). For the attitude (A) domain, data coding for all constructs, except the cue to action, used a 1 to 5 scale (Strongly Disagree = 1, Strongly Agree = 5). The cues to action coding were done using a 1 to 3 scale (Yes = 3, Not Sure = 2 and No = 1). Lastly, the behaviour (B) domain was coded using a 1 to 5 scale (Never = 1 and Always = 5). Total knowledge and attitude scores were calculated and categorised as per Bloom’s cut-off point, a commonly used percentage-based threshold in KAB studies [35] that classifies KAB model domains into three categories: high, moderate and low [36], as detailed in Table 2.

### 2.5. Study Population and Participant Recruitment

A suitable sample size is critical to achieving a stable, representative and accurate statistical model. For structural equation modelling (SEM), it is recommended to have at least ten participants per item (parameter) to achieve a stable model [37,38]. Given that the tool included a total of 125 items (parameters), a minimum sample of 1250 participants was required.

A stratified random sample approach was employed to guarantee adequate representation across various faculties, academic years and disciplines at Yarmouk University, Jordan. The inclusion criteria for this study were as follows: (i) being enrolled as an undergraduate student and (ii) willing to provide informed consent to participate in the study.

The study sample consisted of undergraduate students who were registered at Yarmouk University, Jordan, at the time of the study. Eligible participants were identified and recruited through electronic channels and platforms provided by the University. The recruitment method entailed initiating an email invitation containing a hyperlink to the anonymous questionnaire, supplemented by a minimum of three reminders to enhance the response rate. Additionally, direct recruitment tactics were utilised, involving the approach and invitation of students to engage in various campus activities and events.

### 2.6. Data Collection Procedures

The finalised tool was electronically distributed using the Qualtrics XM platform. Eligible participants were sent an email invitation containing a hyperlink to access the questionnaire. The survey was anonymous, and no personal data or identifiers were collected. Three reminders were sent to improve the response rate. Data collection spanned four months, commencing on 3 January 2021 and concluding on 31 May 2021.

### 2.7. Data Analysis

Completed questionnaires were extracted and logged into an Excel^®^ workbook (Microsoft Office MS, 2013). Prior to analysis, the data were cleaned and coded.

Knowledge, attitude and behaviour constructs were coded as per Table 1. Data were initially analysed descriptively using frequencies, percentages, standard deviation and Z-tests when applicable; a *p*-value < 0.05 was considered statistically significant. A chi-square test of independence was performed to examine the relation between faculty cluster and knowledge, attitude and behaviour scores. To account for potential confounding, participant demographics and characteristics were included in the descriptive and inferential analysis.

The associations between knowledge-related constructs, attitude-related constructs and behaviour-related constructs were examined using the Lavaan package (latent variable analysis), which works under the R environment, to test the model using structural equation modelling (SEM) analysis with latent variables. Analysis of associations between constructs and variables of the proposed model started by assessing the model’s global fit, followed by assessing coefficients and associations. Data was analysed using the Statistical Package for Social Sciences (SPSS) (Version 27, launched in June 2020). SEM reduced potential demographic bias by modelling latent constructs and their measurement error. The stability and suitability of the proposed model were assessed using several fit indices. Following Hu and Bentler (1999) and Kline’s (2016) guidance [38,39], a comparative fit index (CFI) and Tucker–Lewis index (TLI) ≥ 0.90, root mean square error of approximation (RMSEA) ≤ 0.08, and standardised root mean square residual (SRMR) ≤ 0.08 were considered indicative of acceptable and stable model fit.

### 2.8. Ethical Considerations and Approvals

This study was reviewed and approved by the Institutional Review Board at Jordan University of Science and Technology and King Abdulla University Hospital, Irbid, Jordan (reference number: 119/12/2020), on 2 September 2020. Before participating in this study, participants received an information letter at the end of September and were asked to indicate electronic consent before accessing and proceeding with the questionnaire; participants who did not consent to participate were unable to proceed.

## 3. Results

### 3.1. Sample Characteristics

In total, 1375 undergraduate students provided completed surveys. Table 3 summarises the participants’ characteristics and knowledge, attitude and behaviour scores. From the total sample, 911 (66.3%) were females, and more than half (56.4%) were students at humanitarian faculties. Only sixteen participants (1.2%) were involved in an active relationship (married, engaged or in a relationship).

Analysis of COVID-19-related knowledge, attitudes and behaviour (KAB) scores showed that only eighteen (1.3%) had a good knowledge of COVID-19 symptoms, treatment and transmission. On the other hand, 805 (58.5%) participants exhibited good behaviour, whereas 253 (31.4%) reported full compliance with all recommended precautionary behaviours. The chi-square analysis showed that KAB scores were statistically different between the three faculties’ clusters. Lastly, a comparison between female and male participants showed that female participants were significantly more likely to behave positively toward COVID-19 than male participants (*p*-value = 0.02).

### 3.2. Model Global Fit Indices

Global fit indices are statistical tests that assess the stability and adequacy of the retrieved data’s structural equation model (SEM) by assessing how well the model represents the underlying data structure. Among the main global fit indices are the (i) root mean square error of approximation (RMSEA), (ii) comparative fit index (CFI), (iii) standardised root mean square residual (SRMR), (iv) *χ*^2^/*df* ratio and (v) the Tucker–Lewis index (TLI). These indices can help in evaluating the proposed model’s stability, providing guidance for its accuracy or confirming the model’s validity if the indices suggest a satisfactory match [38,40]. As there is no agreement between scholars regarding the cutoff criteria, it is advisable to use several fit indices simultaneously [41]. Moreover, the sample size and the model complexity should be considered when assessing the model fit indices [39,40]. Generally, a model is considered to fit better when the values of CFI and TLI are close to (1) and the values of RMSEA and SRMR are close to (0) [38,40]. In this study’s model, the RMSEA of (0.05) and SRMR value of (0.06) values indicated a good fit for the model.

### 3.3. Structural Equation Model Analysis Output

#### 3.3.1. The Relationship Between Mediator and Endogenous Variables

**Figure 2:**
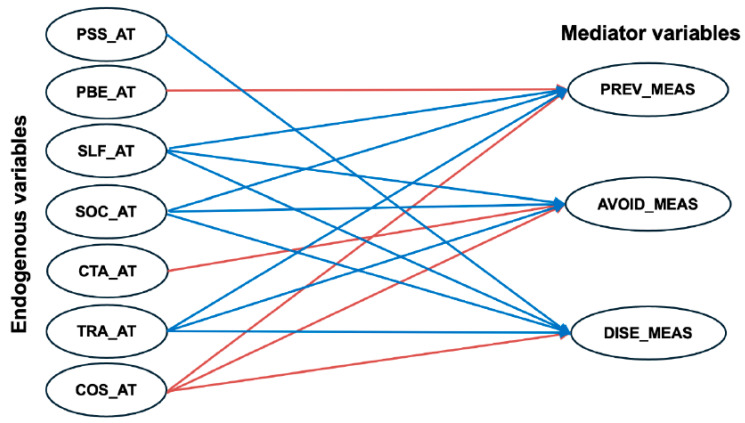
The statistically significant path coefficient relationships between the endogenous variables (behaviours) and the mediator variables (attitudes); red indicates negative associations, while blue indicates positive associations.

As the model was proven to fit the data, the coefficients and relationships between the constructs were assessed. Table 4 shows the path regression coefficients between the constructs for the suggested model. Analysis showed that adopting preventive behaviours was significantly associated with the attitudinal constructs of perceived efficacy of behaviour, perceived self-efficacy, social pressure, trust in authority and the perceived cost (*p*-value < 0.05). Avoidance behaviours were significantly associated with perceived self-efficacy, social pressure, cue to action, trust in authority and the perceived cost of behaviour, see Table 4. Lastly, all attitudinal constructs significantly influenced COVID-19 awareness measures and behaviours except for the perceived efficacy of behaviour and cue to action constructs, see Table 4. The significant associations between attitudinal and behavioural constraints could be used to predict behaviours related to COVID-19, see Table 4 and Figure 2.

#### 3.3.2. The Relationship Between Mediator and Exogenous Variables

**Figure 3:**
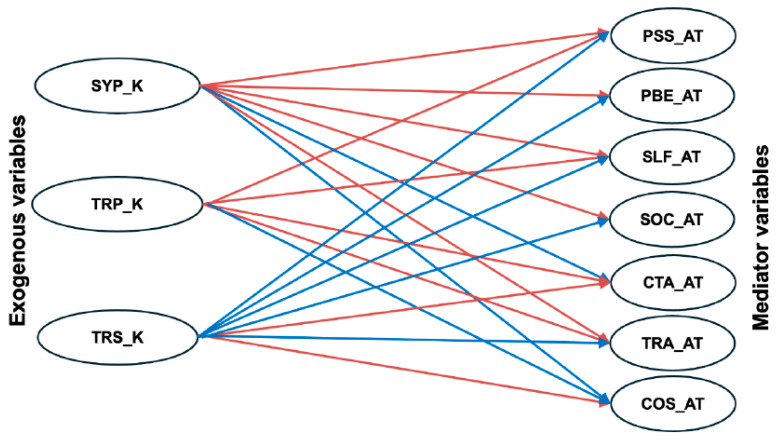
The statistically significant path coefficients between the exogenous variables (knowledge) and the mediator variables (attitudes); red indicates negative associations, while blue indicates positive associations.

The associations between the exogenous variable constructs (knowledge) and the mediator variable constructs (attitude) showed that all knowledge constructs significantly influenced all attitudinal constructs except for perceived self-efficacy and social pressure, which were not influenced by COVID-19 treatment knowledge, see Table 4 and Figure 3.

## 4. Discussion

Using a validated tool, this study evaluated undergraduate students’ knowledge, attitudes, and behaviours on COVID-19 hygiene and preventive measures, examining associations between knowledge, attitude, and behaviour.

The demographic characteristics of the study’s population, where the majority were female and enrolled in humanitarian faculties, were comparable to the national-level gender and discipline composition [42,43]. In theory, demographic characterisation is a key determinant of health behaviours, as suggested by health behaviour scholars and theorists, where factors such as gender and educational level and background influence self-efficacy, behavioural control and normative beliefs [22].

Females are more likely to have a higher health literacy coupled to a more positive attitude and/or a higher compliance with healthy behaviours and practices [44,45,46]. Emerged evidence confirmed gender-based differences, as female respondents were significantly more inclined to adopt positive COVID-19-related behaviours than males (*p*-value = 0.02). These findings emphasise the importance of demographic and social characteristics in guiding and shaping individuals’ knowledge, attitudes and behaviours. Additionally, policymakers should take into consideration the impact of demographic and societal factors in designing health promotion and awareness programmes.

The observed significant differences in knowledge, attitudes and behaviours among students from three different fields highlight variability in health literacy and adherence to behavioural guidelines based on academic discipline. Participants from health and science faculties exhibited a higher understanding of COVID-19 symptoms, transmission and treatment and significantly lower negative attitude towards COVID-19 preventive measures compared to humanities students. This is consistent to the findings of other studies, suggesting that health science students typically had a better understanding of health-related matters and were therefore more likely and willing to comply with recommended health behaviours [47,48]. The observed differences between disciplines could be attributed to the academic curricula’s nature and components, as health science academic programmes strongly emphasise public health measures for prevention [49,50]. Conversely, students enrolled in scientific and humanitarian faculties exhibited diminished levels of knowledge and less uniform attitudes. These findings align with previous studies documenting reduced participation in health-promoting behaviours among non-health students [51,52,53,54,55], highlighting the need for customised educational interventions that target specific disciplines and cultivate favourable attitudes in various academic fields. This approach will be crucial to ensure that all students, regardless of their chosen field of study, possess the necessary skills to engage in effective health-promoting behaviours during public health emergencies [56,57,58,59,60,61]. Despite the data collection period being approximately one year after the COVID-19 outbreak, which was a period characterised by an extensive awareness campaign related to COVID-19 led by the WHO, the Jordanian Ministry of Health and public-health-related agencies [62,63], the results were worrisome, as almost 90% of the study population had a low level of knowledge related to SARS-CoV-2 transmission, symptoms and treatment. The observed lack of knowledge indicates that despite widespread public health campaigns and abundant information across many media platforms, there remained a significant disparity in students’ comprehension of COVID-19 symptoms, treatment and viral transmission dynamics. This knowledge gap aligns with previous research that highlighted comparable patterns in younger demographics, wherein misinformation and conflicting information from many sources can result in confusion and poor understanding of basic principles [64,65].

Despite low knowledge levels, most participants showed positive behaviours, with over one-third adhering to recommended preventive, avoidance, and disease awareness-seeking behaviours. This contradicts the idea that knowledge is the primary catalyst for health-protective behaviours, suggesting that other factors like societal standards and confidence in authority could also impact behaviour [66]. The Health Belief Model (HBM) suggests that individuals are more likely to adopt health-promoting behaviours when they perceive themselves as at-risk, have confidence in the recommended actions, and feel capable of performing them [66]. Health behaviour scholars argue that authoritative mandates and restrictions are more influential in guiding health behaviour than knowledge and awareness [15,22,66,67].

The analysis supports the hypothesis of a connection between COVID-19 knowledge, attitudes, and behaviours. It demonstrates that knowledge significantly influences attitudes, which in turn influences behaviours. This aligns with the KAB model, which suggests that understanding a health issue shapes attitudes [68,69].

Moreover, the significant associations between knowledge- and attitude-related constructs that emerged from this study echoed the findings of earlier studies, albeit in different contexts [32], including that an increased understanding of disease frequently results in more favourable attitudes towards preventive measures, subsequently promoting the adoption of protective behaviours [47,50]. Increased knowledge about COVID-19 symptoms, treatment and transmission is strongly linked to more positive attitudes towards preventive behaviours. The linkage includes trusting authority and the perceived self-efficacy to take action, which are essential factors in promoting health-protective behaviours during pandemics [70,71].

Furthermore, the substantial path coefficients linking attitudes and behaviours emphasise the mediating function of attitudes in the KAB model. The role of attitudes as a mediator between knowledge and action has been extensively studied in health behaviour research [22,72]. In the COVID-19 context, there is a substantial correlation between good attitudes towards preventative behaviours, such as perceiving that wearing masks and maintaining social distance are effective, and practising these behaviours [56,57,58,59,60]. The analysis provides evidence for the proposed correlation between knowledge and behaviour, mediated by attitudes. This finding emphasises the need to improve knowledge and attitudes to promote behaviour change. This notion is strongly supported by the HBM and the theory of planned behaviour [22,25]. These models highlight that although knowledge is fundamental, the attitudes, influenced by perceived risks, benefits and self-efficacy, ultimately dictate whether individuals participate in health-promoting behaviours.

The study found that perceived susceptibility and severity were not significantly associated with any behavioural or knowledge construct, possibly due to a low knowledge level of COVID-19 symptoms, transmission, and treatment. Accurate assessment of the likelihood of acquiring COVID-19 requires understanding the disease symptoms, complications, and transmission mode [32,73,74,75,76,77].

This study comprehensively assessed COVID-19-related knowledge, attitudes, and behaviours of students from various academic fields, and it investigated the associations between the KAB model’s domain using structural equation modelanalysis, which provided evidence of mediational mechanisms that underline behavioural adherence and compliance. The study’s sample size allows for a reliable analysis of the factors influencing students’ reactions to the pandemic. Moreover, the involvement of students from various faculties provided valuable insights into how educational contexts impact health-related behaviours.

However, the study has several limitations and shortcomings related to its design, sampling and conduct. First, the study’s cross-sectional design limits its ability to establish causal relationships. Additionally, the study did not consider the influence of external factors such as media, government regulations, or the different stages of the pandemic. Second, the sampling framework and the study population, Yarmouk University students, might hinder and limit the possibility of result generalisability and evidence transferability. Third, the reliance on the self-administration approach in data collection could introduce social desirability and recall bias, which could have affected participants’ responses and feedback. Therefore, caution should be practised when interpreting and reporting the findings of this study.

## 5. Conclusions

The findings highlight the urgent need for customised health education and communication strategies that address the unique needs and attributes of different academic fields. Health students could benefit from specialised training, while scientific and humanitarian students require more fundamental instruction on preventative measures and risk perceptions [50]. Health policymakers should collaborate with educational institutions to create adaptive learning environments that accommodate the changing needs of students from various academic backgrounds, emphasising the issue’s urgency [70]. Additionally, the study confirmed the proposed sequential influence between knowledge, attitudes and behaviours, which is consistent with the KAB model. The reported gender- and discipline-based differences emphasised the role of context in shaping individuals’ attitudes and behaviours.

## Figures and Tables

**Table 1:** Domain, constructs and scales in the data collection tool.

Domain *	Construct	Details	
Knowledge (Exogenous Variable)	Symptoms Knowledge	Definition	The awareness and understanding of COVID-19 signs and symptoms
		No. of Items ^†^	9
		Code	SYP_K
		Used Scale	True, False, Do Not Know
	Treatment Knowledge	Definition	The comprehension and awareness an individual has about different approaches and interventions available to treat and manage COVID-19
		No. of Items ^†^	10
		Code	TRP_K
		Used Scale	True, False, Do Not Know
	Transmission Knowledge	Definition	The awareness and understanding of how COVID-19 transmits from one person to another or from the environment to a person
		No. of Items ^†^	12
		Code	TRS_K
		Used Scale	True, False, Do Not Know
Attitude (Mediator Variable)	Perceived Susceptibility and Severity	Definition	The perception about the likelihood of acquiring COVID-19 and being infected
		No. of Items ^†^	7
		Code	PSS_AT
		Used Scale	Strongly Disagree, Disagree, Neutral, Agree, Strongly Agree
	Perceived Efficacy of Behaviour	Definition	The perceived efficacy of a specific action or behaviour in attaining a COVID-19-related outcome
		No. of Items ^†^	13
		Code	PBE_AT
		Used Scale	Strongly Disagree, Disagree, Neutral, Agree, Strongly Agree
	Perceived Self-efficacy	Definition	The perception of the ability to carry out and perform COVID-19 preventive and avoidance practices
		No. of Items ^†^	13
		Code	SLF_AT
		Used Scale	Strongly Disagree, Disagree, Neutral, Agree, Strongly Agree
	Social Pressure	Definition	The perception regarding the expectations of the closed social cycle or authorities of an individual’s behaviour to avoid COVID-19
		No. of Items ^†^	14
		Code	SOC_AT
		Used Scale	Strongly Disagree, Disagree, Neutral, Agree, Strongly Agree
	Cues to Action	Definition	The environmental or self-based stimuli motivating an individual to engage in a certain behaviour linked to COVID-19
		No. of Items ^†^	12
		Code	CTA_AT
		Used Scale	Yes, No, Unsure
	Trust in Authority	Definition	The perception regarding authority openness and transparency in communications related to COVID-19
		No. of Items ^†^	4
		Code	TRA_AT
		Used Scale	Strongly Disagree, Disagree, Neutral, Agree, Strongly Agree
	Perceived Cost	Definition	The perception regarding practical barriers to COVID-19-related behaviours, such as financial cost and time constraints
		No. of Items ^†^	15
		Code	COS_AT
		Used Scale	Strongly Disagree, Disagree, Neutral, Agree, Strongly Agree
Behaviour (Endogenous Variable)	Avoidance Behaviour	Definition	The deliberate acts and tactics that individuals adopt in order to avert contact with the SARS-CoV-2 virus, which is responsible for causing COVID-19
		No. of Items ^†^	8
		Code	AVO_BEH
		Used Scale	Never, Rarely, Sometimes, Often and Always
	Preventive Behaviour	Definition	Measures implemented to minimise the spread of the SARS-CoV-2 virus, responsible for causing COVID-19
		No. of Items ^†^	5
		Code	PRE_BEH
		Used Scale	Never, Rarely, Sometimes, Often and Always
	Disease Awareness Behaviour	Definition	The acts and practices individuals adopt to improve their understanding of COVID-19 and effectively reduce the risk of infection and transmission
		No. of Items ^†^	TRT_BEH
		Code	3
		Used Scale	Never, Rarely, Sometimes, Often and Always

COVID-19: coronavirus disease of 2019; SARS-CoV-2: severe acute respiratory syndrome coronavirus 2. * Domains within the knowledge, attitude and behaviour model. ^†^ Reflects number of statements within each construct; total number is 125 items.

**Table 2:** Bloom’s cut-off categories of the total knowledge and attitude scores.

Domain	Category *	Total Score	(%)
Knowledge (K)	High	25–31 out of 31	80–100%
	Moderate	19–24 out of 31	60–79%
	Low	<19 out of 31	<60%
Attitude (A)	Positive	293–366 out 366	80–100%
	Neutral	220–292 out of 366	60–79%
	Negative	<220 out of 366	<60%
Behaviour (B)	Good	64–80 out of 80	80–100%
	Fair	48–63 out of 80	60–79%
	Poor	<48 out of 80	<60%

* As per Bloom’s cut-off points. % Percentage.

**Table 3:** Participants characteristics and knowledge, attitude and behaviour scores.

Attributes	Participants’ Group		
	Health Faculties ^1^ N (%)	Scientific Faculties ^2^ N (%)	Humanitarian Faculties ^3^ N (%)
**Demographics and characteristics**			
**Gender**			
Female	182 (67.2%)	201 (61.3%)	528 (68.0%)
Male	85 (31.4%)	115 (35.1%)	227 (29.3%)
Prefer not to say	4 (1.5%)	12 (3.7%)	21 (2.7%)
**Age**			
Average (±SD)	20.4 (±3.8)	19.4 (±1.51)	19.6 (±2.78)
**Marital Status**			
Single	266 (98.2%)	318 (97.0%)	755 (97.3%)
Married	2 (0.7%)	4 (1.2%)	10 (1.3%)
Prefer not to say	3 (1.1%)	6 (1.8%)	11 (1.4%)
**Knowledge, attitude and behaviour scores**			
Knowledge *			
High	10 (3.7%)	3 (0.9%)	5 (0.6%)
Moderate	51 (18.8%)	32 (9.8%)	47 (6.1%)
Low	210 (77.5%)	293 (89.3%)	724 (93.3%)
**Attitude ^†^**			
Positive	42 (15.5%)	61 (18.6%)	146 (18.8%)
Neutral	216 (79.7%)	230 (70.1%)	558 (71.9%)
Negative	13 (4.8%)	37 (11.3%)	72 (9.3%)
**Behaviour ^‡^**			
Good	144 (53.1%)	178 (54.3%)	483 (62.2%)
Fair	98 (36.2%)	105 (32.0%)	221 (28.5%)
Poor	29 (10.7%)	45 (13.7%)	72 (9.3%)

**N**: number; **SD**: standard deviation. **^1^** Health Faculties: (i) Faculty of Medicine and (ii) Faculty of Pharmacy. **^2^** Scientific Faculties: (i) Faculty of Science, (ii) Faculty of Hijjawi for Engineering Technology and (iii) Faculty of Information Technology and Computer Science. **^3^** Humanitarian Faculties: (i) Faculty of Arts, (ii) Faculty of Business, (iii) Faculty of Shari’a and Islamic Studies, (iv) Faculty of Law, (v) Faculty of Educational Sciences, (vi) Faculty of Physical Education and Sport Sciences, (vii) Faculty of Archaeology and Anthropology, (viii) Faculty of Mass Communication and (ix) Faculty of Tourism and Hotel Management. * Chi-square statistic (X^2^) is 55.01, degrees of freedom = 4, *p*-value < 0.00001. ^†^ Chi-square statistic (X^2^) is 10.80, degrees of freedom = 4, *p*-value = 0.03. ^‡^ Chi-square statistic (X^2^) is 12.50, degrees of freedom = 4, *p*-value = 0.01.

**Table 4:** Path regression coefficients between the constructs for the suggested model.

	Construct		Est.	S.E	Z.-Value	*p*-Value	Std.All
The Behaviour-Attitude Relationship (Endogenous and Mediator Variables)	Preventive Behaviours	PSS_AT	0.0018	0.0183	0.0968	0.9229	0.0025
		PBE_AT	−0.0742	0.0363	−2.0435	0.0410	−0.0760
		SLF_AT *	0.4942	0.0417	11.8654	0.0000	0.4972
		SOC_AT *	0.1570	0.0348	4.5056	0.0000	0.1616
		CTA_AT	−0.0414	0.0304	−1.3644	0.1724	−0.0314
		TRA_AT *	0.1103	0.0162	6.8140	0.0000	0.1847
		COS_AT *	−0.1471	0.0288	−5.0996	0.0000	−0.1221
	Avoidance Behaviours	PSS_AT	0.0335	0.0226	1.4817	0.1384	0.0377
		PBE_AT	0.0749	0.0445	1.6823	0.0925	0.0625
		SLF_AT *	0.3246	0.0479	6.7823	0.0000	0.2656
		SOC_AT *	0.2627	0.0432	6.0868	0.0000	0.2200
		CTA_AT	−0.1368	0.0376	−3.6340	0.0003	−0.0843
		TRA_AT *	0.1235	0.0198	6.2306	0.0000	0.1682
		COS_AT *	−0.1520	0.0350	−4.3417	0.0000	−0.1026
	Disease Awareness Behaviours	PSS_AT	0.0602	0.0304	1.9787	0.0478	0.0538
		PBE_AT	−0.0767	0.0600	−1.2792	0.2008	−0.0507
		SLF_AT *	0.4726	0.0640	7.3811	0.0000	0.3063
		SOC_AT *	0.2436	0.0574	4.2452	0.0000	0.1616
		CTA_AT	−0.0682	0.0504	−1.3534	0.1759	−0.0333
		TRA_AT *	0.2178	0.0266	8.1832	0.0000	0.2349
		COS_AT *	−0.2558	0.0477	−5.3626	0.0000	−0.1368
The Attitude–Knowledge Relationship (Mediator and Exogenous Variables)	Perceived Susceptibility and Severity	SYP_K *	−1.3319	0.3467	−3.8416	0.0001	−0.2819
		TRP_K *	−0.5898	0.1636	−3.6047	0.0003	−0.1355
		TRS_K *	2.7356	0.3597	7.6045	0.0000	0.6160
	Perceived Efficacy of Behaviour	SYP_K *	−2.8605	0.3854	−7.4218	0.0000	−0.8187
		TRP_K	−0.0484	0.1465	−0.3302	0.7412	−0.0150
		TRS_K *	4.6951	0.4574	10.2641	0.0000	1.4295
	Perceived Self-efficacy	SYP_K *	−3.4048	0.4223	−8.0621	0.0000	−0.9932
		TRP_K	−0.3715	0.1595	−2.3291	0.0199	−0.1176
		TRS_K *	4.8184	0.4726	10.1954	0.0000	1.4952
	Social Pressure	SYP_K *	−3.2212	0.4078	−7.8993	0.0000	−0.9184
		TRP_K	−0.1603	0.1527	−1.0501	0.2937	−0.0496
		TRS_K *	4.7486	0.4622	10.2733	0.0000	1.4402
	Cues to Action	SYP_K *	0.4677	0.1725	2.7120	0.0067	0.1812
		TRP_K *	−0.4792	0.0924	−5.1832	0.0000	−0.2015
		TRS_K *	−0.6067	0.1543	−3.9321	0.0001	−0.2500
	Trust in Authority	SYP_K *	−3.1964	0.4962	−6.4417	0.0000	−0.5603
		TRP_K *	−0.9933	0.2267	−4.3814	0.0000	−0.1890
		TRS_K *	4.6562	0.5157	9.0282	0.0000	0.8683
	Perceived Cost	SYP_K *	0.7544	0.1966	3.8362	0.0001	0.2668
		TRP_K *	0.5636	0.1070	5.2686	0.0000	0.2164
		TRS_K *	−0.8534	0.1785	−4.7801	0.0000	−0.3211

* *p*-value < 0.05. COS_AT: perceived cost; CTA_AT: cues to action; Est: estimate; PBE_AT: perceived efficacy of behaviour; PSS_AT: perceived susceptibility and severity; *p*-value: probability value; S.E: standardised error; SLF_AT: perceived self-efficacy; SOC_AT: social pressure; SYP_K: symptoms knowledge; Std.All: path standardised regression coefficient; TRA_AT: trust in authority; TRP_K: treatment knowledge; TRS_K: transmission knowledge.

## Data Availability

Research data will be available upon request to the corresponding author Saja A. Alnahar.

## Abbreviations

A	Attitude
CHERRIES	Checklist for Reporting Results of Internet E-Surveys
CFI	Comparative Fit Index
COVID-19	Coronavirus Disease of 2019
B	Behaviour
HBM	Health Belief Model
HEI	Higher Education Institution
K	Knowledge
KAB	Knowledge, Attitude and Behaviour model
MoH	Ministry of Health
RMSEA	Root Mean Square Error of Approximation
SARS-CoV-2	Severe Acute Respiratory Syndrome Coronavirus 2
SEM	Structural Equation Model
SRMR	Standardised Root Mean Square Residual
TLI	The Tucker–Lewis Index
WHO	World Health Organisation
